# Microwave aided conversion of cellulose to glucose using polyoxometalate as catalyst†

**DOI:** 10.1039/d1ra04426e

**Published:** 2021-10-26

**Authors:** Manami Nakamura, Md. Saidul Islam, Mohammad Atiqur Rahman, Rabin Nurun Nahar, Masahiro Fukuda, Yoshihiro Sekine, Jorge N. Beltramini, Yang Kim, Shinya Hayami

**Affiliations:** Department of Chemistry, Graduate School of Science and Technology, Kumamoto University 2-39-1 Kurokami, Chuo-ku Kumamoto 860-8555 Japan hayami@kumamoto-u.ac.jp; Institute of Industrial Nanomaterials (IINa), Kumamoto University 2-39-1 Kurokami, Chuo-ku Kumamoto 860-8555 Japan; Priority Organization for Innovation and Excellence, Kumamoto University 2-39-1 Kurokami, Chuo-ku Kumamoto 860-8555 Japan; Centre for Tropical Crops and Bio-Commodities, Queensland University of Technology Brisbane 4000 Australia; International Research Center for Agricultural and Environmental Biology (IRCAEB) 2-39-1 Kurokami, Chuo-ku Kumamoto 860-8555 Japan

## Abstract

The viability of biorefining technology primarily depends on the facile cellulose conversion route with adequate conversion efficiency. Here we have demonstrated the microwave-assisted hydrolysis of cellulose to glucose using polyoxometalate (POM) clusters as acid catalysts. Two different types of POM, including Wells–Dawson and Keggin were justified as catalysts in the cellulose conversion process. In particular, the cellulose to glucose catalytic conversion using Wells–Dawson type POMs has not been reported to date. Also, even though there have been some previous reports about the catalytic biomass conversion of Keggin type POMs, the systematic study to optimize the conversion efficiency in terms of catalyst amount, reaction temperature, reaction time, and the amount of solvent is lacking. Under the experimental conditions employed, the Keggin-type catalyst showed higher cellulose conversion and glucose yield than the Wells–Dawson-type catalyst. Furthermore, the cellulose conversion efficiency and glucose yields were optimized by tuning the reaction conditions including temperature, reaction time, and the amount of solvent. Under optimized conditions, the Keggin-type POM catalyst shows a remarkably high glucose yield of 77.2% and a cellulose conversion of 90.1%. The unique complex properties of the POM catalyst, including being (i) strong acids with extremely high Brønsted and Lewis acidity and (ii) efficient microwave adsorbants which enhanced interaction between substrate and the catalyst can be attributed to the outstanding efficacy of the conversion process.

## Abstract 2

The polyoxometalate (POM) catalysed conversion of cellulose to glucose is demonstrated. A remarkably high cellulose conversion of 90.1% with a glucose yield of 77.2% were achieved.

## Graphical abstract

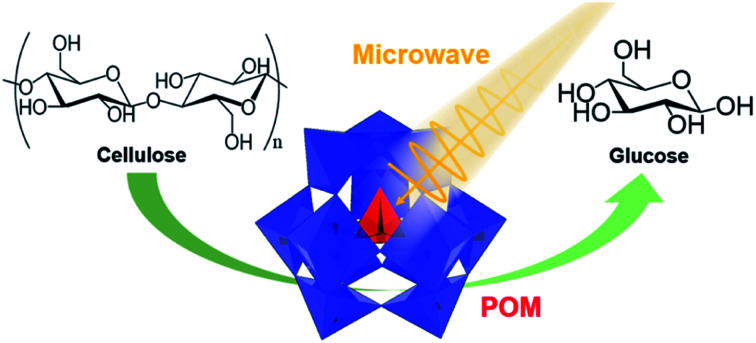

## Introduction

The utilization of abundant, low-cost and environment-friendly lignocellulosic biomass-derived fossil resources to replace the state-of-art petroleum-based chemicals is one of the key research targets and has been the focus of researchers worldwide during the last decades. The sustainable bio-economy concept consisting of achieving fuels, chemicals and materials from biomass using an integrated biorefinery route has attained much interest; these renewable energy resources and solving environmental concerns about carbon dioxide emissions are important. However, the low yields of the desired products resulting from very complex reaction processes along with many parallel reactions during the biomass conversion process largely limit the expected outcome of the biomass conversion process.^1,2^ Therefore, the designing of a facile cellulose conversion route along with justifying the optimum reaction parameters to maximize the yields of the target products is a key aim for biomass conversion research.

Lignocellulosic biomass is estimated to contain 40–50% cellulose, 20–40% hemicellulose, and 20–30% lignin, which indicates the importance of the efficient valorization of cellulose to make biomass a sustainable energy source.^3,4^ Cellulose is composed of glucose units linked by β-1,4-glycosidic bonds (or acetal bonds) to create long chains. The linear structure of the cellulose chain forms intra- and intermolecular hydrogen bonds which lead to the chains becoming microfibrils with both crystalline regions and areas of less ordered or amorphous cellulose. The crystal structure and hydrogen bonding in cellulose greatly limit the access of reactants and catalysts to β-1,4-glycosidic bonds. Efficiently disrupting hydrogen bonding, making many more β-1,4-glycosidic bonds accessible to reactants and catalysts, is critical to increasing the cellulose hydrolysis rate. On the other hand, monomeric carbohydrates such as glucose are the most important materials that can be directly used as feedstock for the production of bio-based chemicals.^5^ The difficulty of efficiently hydrolyzing recalcitrant crystalline cellulose has proven to be a major barrier to cellulose being extensively used in bio-energy production.^6^

Currently, several available acid-catalyzed technologies are mainly adopted for the hydrolysis of biomass that includes the use of dilute acid,^7^ concentrated acid,^8^ and organic acid.^9,10^ However, the limitations in terms of product separation, corrosion, high cost and difficulties in recycling, environmental concerns of waste effluents, make these processes unattractive. Sasaki and co-workers^11^ found that cellulose is rapidly dissolved and depolymerized in supercritical water at 300–320 °C without acid. They demonstrate that supercritical water completely disrupts the intramolecular and intermolecular hydrogen-bond linkages. Enzymatic hydrolysis^12^ is another interesting hydrolysis route but suffers from the low hydrolysis efficiency and high cost of the enzyme. Therefore, research interests are persisted on the facile hydrolysis of cellulose with adequate conversion efficiency that can make a significant change in the utilization of only globally available, renewable, and abundant biomass-derived carbon-neutral sources.

Polyoxometalates (POMs) are composed of cations and polyanion clusters and are known to be environmentally friendly and economically viable catalysts for homogeneous and heterogeneous acid catalyzed reactions. The unique physical and chemical properties including strong Brønsted acidity, high proton mobility, high solubility in water and various oxygen-containing organic solvents, good stability, and easy regeneration after utilization facilitated the POM based catalyst very attractive in biomass conversion technologies.^13,14^ However, the catalytic activity of the Wells–Dawson type POMs towards cellulose to glucose conversion are not well-reported to date. Also, despite some previous studies on the hydrolytic activity of different POMs have been conducted on crystalline cellulose, and the results have demonstrated the high catalytic activity and environmental friendliness of homogeneous POMs,^15^ micellar POMs,^16^ cesium salt^17^ and metal salts,^18^ the facile route with optimize the reaction conditions to maximize the cellulose conversion and glucose yield using pristine Keggin-type POMS are still remain a challenge. Therefore, in the current study, (i) we have justified the catalytic activity of the Wells–Dawson (acid & salts) type POMs and (ii) the systematic study to optimize the cellulose conversion efficiency and glucose yield using pristine Keggin-type POM catalyst.

On the other hand, among the physicochemical pre-treatment methods, the promoting effect of microwave irradiation on dilute alkali- and acid-catalyzed hydrolysis of cellulosic materials have been well established.^19–21^ Additionally, the dielectric property of the cellulose and aqueous POM solution are interesting for increasing the microwave-absorption capability of the reaction system and facilitate the fast energy transfer that improves the reaction rate.^22,23^ Moreover, the ball milling can efficiently act to alter the crystalline cellulose structure to an amorphous structure and its hydrogen linkage heavily affected the efficiency of hydrolysis. Based on the above advantages, in the current work, we demonstrate highly efficient hydrolysis of cellulose to glucose using a combination of physicochemical pre-treatment (ball milling), POM catalysis, and microwave aided methods. The influence of various reaction parameters including temperature, reaction time, and the amount of substrate were evaluated to estimate the optimum condition for cellulose conversion. The maximum cellulose conversion of 90.1% and glucose yield of 77.2% were achieved with a microwave aided process at 160 °C, 15 reaction time and 3 mL solvent.

## Experimental

We have justified the catalytic biomass conversion from three different POM samples namely the Wells–Dawson salt K_6_[P_2_W_18_O_60_], Wells–Dawson acid H_6_[P_2_W_18_O_60_], and Keggin acid H_3_[PW_12_O_40_]. The former two were prepared in our laboratory by following the procedure described elsewhere and the third one was provided by Aldrich (reagent grade, 99.7%).

### Materials

All reagents utilized in this work were in analytical grade and were used without further purification. Glucose, levulinic acid, fructose and 5-hydroxymethylfurfural standard reagents were purchased from Wako Pure Chemical Industries. Cellulose was purchased from Sigma-Aldrich. H_3_PO_4_, Na_2_WO_4_·2H_2_O, KCl were purchased from Wako Pure Chemical Industries.

### Dawson-type acid synthesis

The Wells–Dawson-type salt K_6_[P_2_W_18_O_60_] was synthesized using previously reported results by Lyon *et al.*^25^ In a typical synthesis route, in a boiling aqueous solution of Na_2_WO_4_·2H_2_O concentrated H_3_PO_4_ was added in a 4 : 1 acid/salt ratio followed by reflux the system for 8 h. The product salt was precipitated by adding KCl, and purified by recrystallization followed by cooling overnight at 278 K. The product, which is a mixture of the α/β K_6_P_2_W_18_O_62_·10H_2_O isomer, was filtered, washed and then vacuum-dried overnight. The Wells–Dawson acid (H_6_P_2_W_18_O_62_·aq.) was prepared by the Drechsel method^24^ from the prepared Wells–Dawson salt by treating with ether and concentrated HCl (37%) solution followed by be separating it from the solution.

### Ball-milling experiments

Ball milling cellulose was prepared as follows: cellulose (5.0 g) was loaded into a pot mill (3.6 L) with an Al_2_O_3_ ball (mass of 2 kg and diameter of 1.5 cm). Ball-milling experiments were performed on an AS ONE ball-mill machine. The spinning speed was set at 280 rpm for 24 h.

### Powder X-ray diffraction method (PXRD)

Powder X-ray diffraction data (PXRD) was collected on a Rigaku MiniFlex II ultra (30 kV/15 mA) X-ray diffractometer using Cu Kα radiation (*λ* = 1.5406 Å) in the 2*θ* range of 5–60°.

### Catalytic activity procedure and product analysis

The microwave-assisted catalytic conversion of cellulose was carried out using microwave reaction vials. In a typical experimental run, 200 mg cellulose, 3 mL solvent and 420 mg catalysts were charged into a 10 mL reactor. After being sealed with a cap, the reactor containing the mixture was mounted in a microwave reactor apparatus (Biotage Initiator+) while set the frequency of 2.45 GHz with out-put power of 400 W and heated at a specified reaction time under magnetic stirring. Time zero of the reaction was defined as the time when the reactor reached its set point temperature. Reactions were performed in triplicate to asses reproducibility of the results. The liquids and solid residues were separated by filtration. HPLC was used to separate and quantify liquid products. Liquid samples were collected after reaction and the concentration of the product species were quantified using Agilent Technologies HPLC with a ZORBAX Eclipse Plus C18 as the analytical column and both RID (refractive index) and VWD (UV-Vis) detectors. The operating condition of the HPLC were: oven temperature – 35 °C, mobile phase – 5 mM H_2_SO_4_; flow rate – 0.6 mL min^−1^; injection volume – 5 μL. The concentrations of glucose, formic acid (FA), levulinic acid (LA) and hydroxymethylfurfural (HMF) were quantified through the external standard method and calibration curves of commercially available standard substrates.Conversion (wt%) = {1 − (unreacted cellulose (mg))/(initial cellulose input (mg))} × 100Product yield (%) = ((moles of C in product)/(moles of C in initial reactant)) × 100

## Results and discussion

**Fig. 1:**
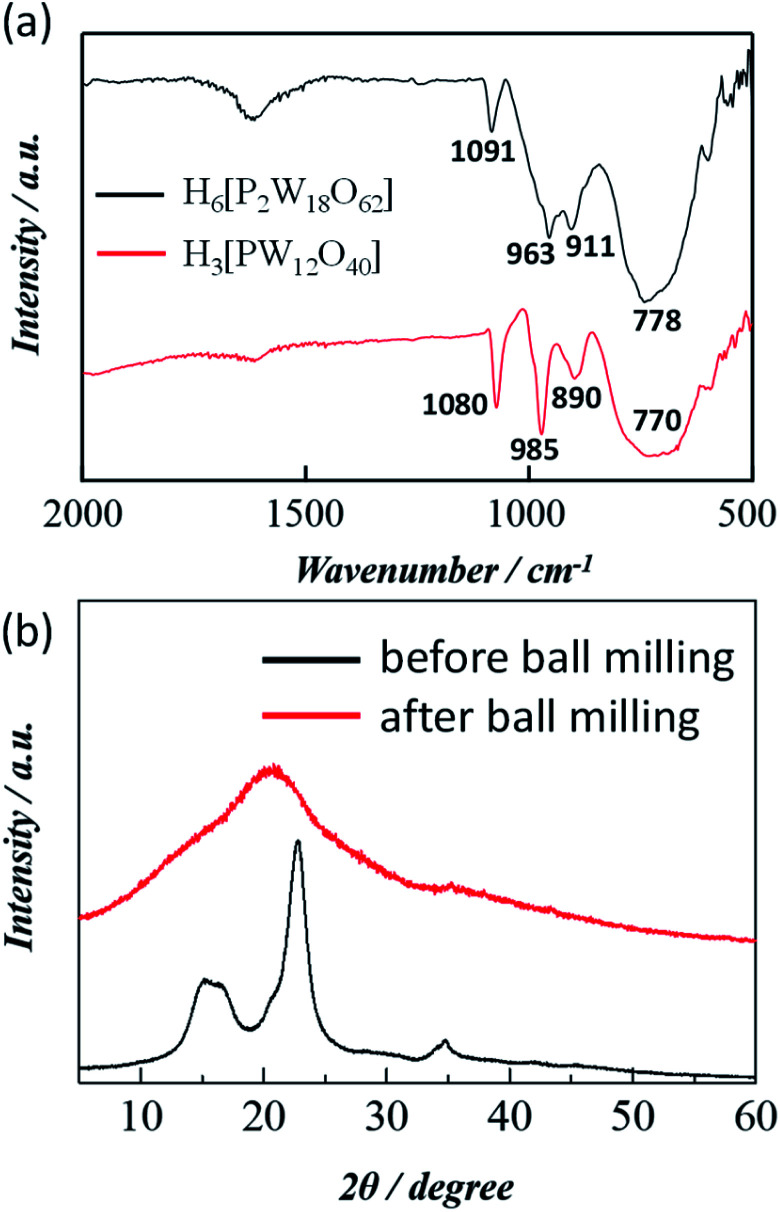
(a) FTIR spectra of H_6_[P_2_W_18_O_62_] and H_3_[PW_12_O_40_] and (b) PXRD pattern of cellulose before and after ball milling.

The formation of Wells–Dawson acid under the experimental conditions employed was confirmed using FTIR spectrum. Fig. 1a compares the FTIR spectra of Wells–Dawson acid H_6_[P_2_W_18_O_60_], and Keggin acid H_3_[PW_12_O_40_] POM catalyst. The strong vibration bands for H_6_[P_2_W_18_O_60_] at 1091, 963, 911 and 778 cm^−1^ can be assigned to the stretching frequency of the PO_4_ tetrahedron, W=O (terminal bonds), inter W–O–W and intra W–O–W bridges, respectively.^26^ On the other hand, the FTIR peaks for Keggin POM observed at 1080, 985, 890, and 770 cm^−1^, corresponding to the asymmetric vibrations of central oxygen (P–O), terminal asymmetric oxygen (W–O), corner shared oxygen (W–O–W), and edge shared oxygen (W–O–W), respectively which is in accord with the previous literature.^27^ X-ray photoelectron spectroscopy (XPS) analysis was conducted to validate the oxidation state of the central metal for Keggin and Wells–Dawson catalyst and the W 4f XPS spectra for both catalysts are shown in Fig. S1.† The binding energy peak around 36.3 eV is ascribed to W 4f_7/2_, while the peak around 38.4 eV is assigned to W 4f_5/2_. The binding energy of 36.3 eV for W 4f_7/2_ core excitation is approximately equal to that of W^6+^, confirming that such stable oxidation state of tungsten could be ascribed both catalysts reported elsewhere.^27^ It is difficult to explain the comparative difference of catalytic performance of Wells–Dawson and Keggin type POM from the characterization data such as FT-IR and XPS rather some previous reports suggest that the higher catalytic activity of the Keggin catalyst is in line with the existence of a higher number of Brønsted acid sites in Keggin catalyst.^18^

Fig. 1b compares the PXRD patterns of cellulose before and after the ball-milling. The PXRD pattern before the treatment showed diffraction peaks of crystalline cellulose around 16°, 22°, and 34°. The strongest peak, at 2*θ* = 22.6°, originates from the cellulose crystalline plane (002).^30,31^ Interestingly, the crystalline peaks disappeared with the appearance of an amorphous broad peak around 20° in the PXRD pattern of the cellulose milled for 24 h indicates an obvious decrease of cellulose crystallinity during ball milling which has been used for the following catalytic experiments. It is well documented that the amorphous part of cellulose is more readily accessible by water and other reactants.^28^ Molecular dynamics simulations by Mazeau *et al.*, demonstrated the total number of hydrogen bonds per repeat units is eight in the crystalline form and is only 5.3 in the amorphous form.^29^ They also showed that the crystalline form of cellulose has much larger cohesive energy density than noncrystalline forms. This suggests that non-crystalline forms of cellulose will be more reactive for hydrolysis. Therefore, here we use the 24 h ball milling driven pre-treated cellulose (cellulose-BM) to increase the non-crystalline fraction that increases the number of accessible β-1,4-glycosidic bonds.

**Fig. 2:**
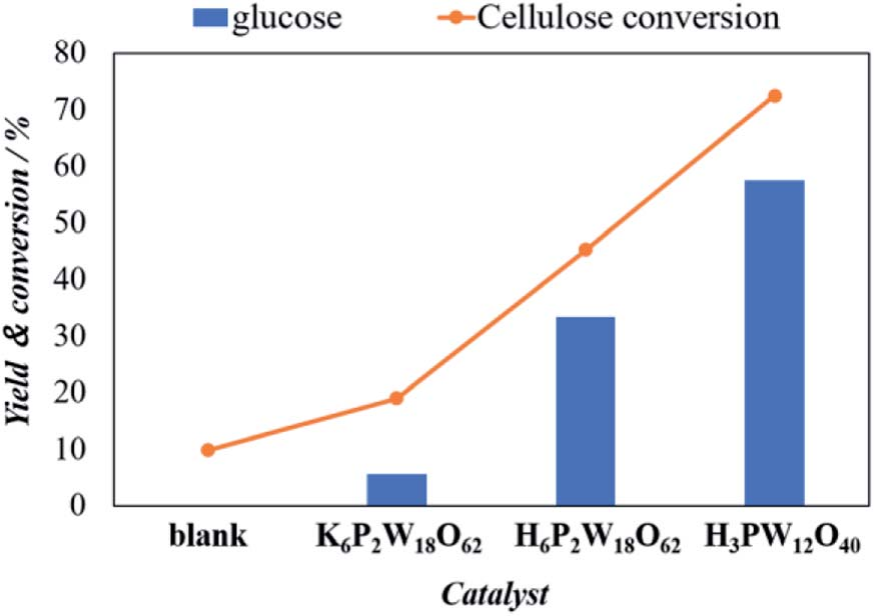
The effects of different POM clusters including K_6_[P_2_W_18_O_62_] (0.09 mmol), H_6_[P_2_W_18_O_62_] (0.10 mmol) and H_3_[PW_12_O_40_] (0.15 mmol) on the hydrolysis of ball milling cellulose.

Among the different types of POMs, Keggin- and Wells–Dawson are viewed as the most classical metal-oxo anionic clusters that might have the ability to act as oxidation catalysts in various reactions including cellulose conversion. Therefore, in this study, we have justified the catalytic performance of the Wells–Dawson (acid & salts) and Keggin type POMs for catalytic cellulose to the glucose conversion reaction. The effects of K_6_[P_2_W_18_O_62_], H_6_[P_2_W_18_O_62_] and H_3_[PW_12_O_40_] POM catalysts for cellulose conversion and glucose yield using cellulose-BM as a substrate, are shown in Fig. 2. The microwave reaction was performed at 160 °C temperature, 5 minutes reaction time, 400 mg catalyst, 200 mg of cellulose and 3 mL of water as the solvent. Clearly, the cellulose conversion and glucose yield show the following order: H_3_[PW_12_O_40_] > H_6_[P_2_W_18_O_62_] > K_6_[P_2_W_18_O_62_] > blank. Among the two Wells–Dawson type POMs containing different cations, both the cellulose conversion and glucose yield were higher in H_6_[P_2_W_18_O_62_] with cellulose conversion of 47.4% and glucose yield of 36.9% than that of K_6_[P_2_W_18_O_62_] which showed cellulose conversion of 18.7% and glucose yield of 7.3%. This observation confirms the proton dissociated in solution is important in the hydrolysis reaction of glucose from cellulose. On the other hand, the Keggin-type POM shows the cellulose conversion of 73.4% along with glucose yield of 57.5%. The higher hydrolysis ability of Keggin-type catalyst is in accord with some previous report where they confirm that it contains a higher number of Brønsted acid sites than that of Dawson-type catalyst.^18,32,33^

**Fig. 3:**
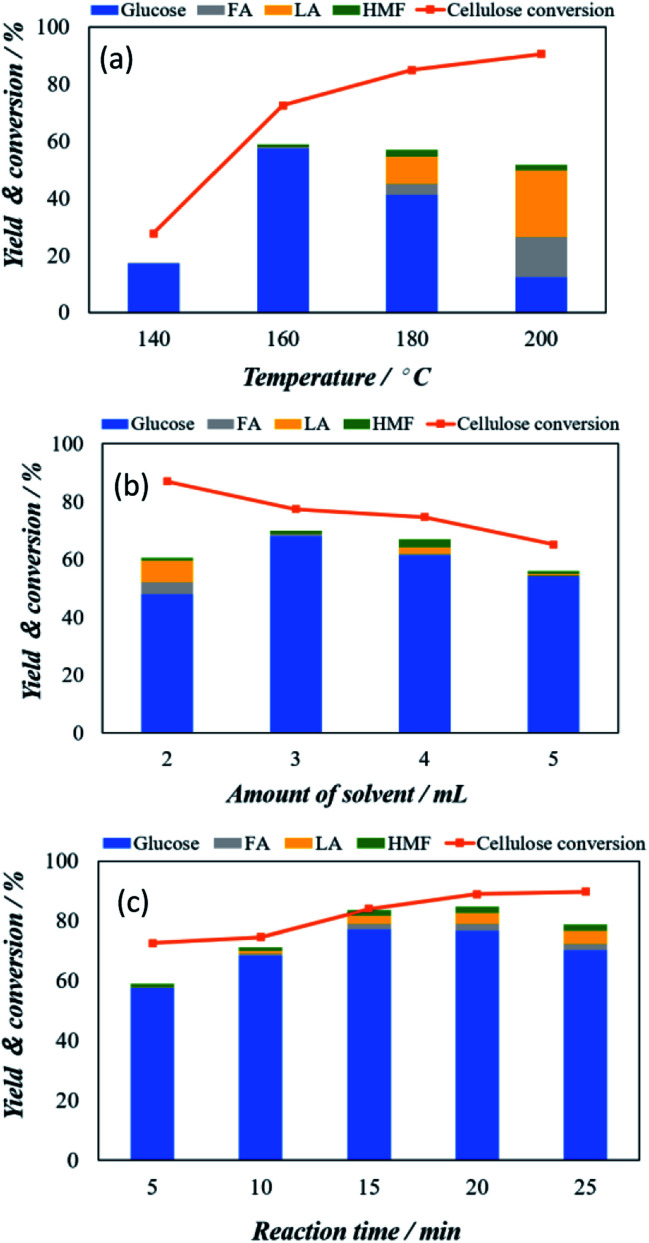
Optimization of cellulose conversion and glucose yield using H_3_PW_12_O_40_ as catalysts (a) effect of reaction temperature (b) amount of solvent used and (c) reaction time employed.

To find the optimum hydrolysis of cellulose with maximum glucose yield, we selected the Keggin type catalyst to further tune the experimental conditions, including temperature, amount of solvent, and reaction time. Fig. 3a shows the temperature dependency of the microwave-assisted POM catalysed cellulose conversion and glucose yield from 140 °C to 200 °C. As can be seen in Fig. 3a, the cellulose conversion shows a linear relation with the increasing temperature and increases from 28.1% at 140 °C to the 91.8% at 200 °C. However, the glucose yield shows a sharp increase from 18.2% at 140 °C to 57.2% at 160 °C and after that gradual decrease to 42.3% at 180 °C and 16.8% at 200 °C. Significantly, at temperature 180 °C and 200 °C the decrease in glucose is associated with the appearance of other chemicals including formic acid (FA), levulinic acid (LA) and HMF which might be attributed to the decomposed of glucose to FA and LA under high temperature condition. Based on the above observation, 160 °C is considered as optimized microwave-assisted heating condition.

At this optimized temperature condition, we have investigated the effect of amount of solvent between 2 to 5 mL and the results obtained is shown in Fig. 3b. The cellulose conversion decreased gradually as the amount of solvent increased. The cellulose conversion is maximum with a value of 86.9% when the amount of solvent was 2 mL, but the yield of glucose was only 48.0%. The glucose yield increased significantly to 67.9% at 3 mL and is decrease again to 62.1% at 4 mL solvent and 57.2% at 5 mL solvent. Therefore, the amount of solvent as 3 mL is the optimum hydrolysis condition. The effect of reaction time from 5 to 25 minutes is investigated under these conditions (160 °C and 3 mL solvent) and is shown in Fig. 3c. With increasing reaction time, the gradual increase in cellulose conversion from 72.2% at 5 min to 92.1% at 25 minutes is observed. However, glucose yield gradually increases from 57.5% at 5 minutes to 77.2% at 15 minutes. The cellulose conversion was 90.1%. With further increase reaction time, glucose yield started to decrease slightly despite the total conversion of cellulose continuously increases.

In addition, it can be noted that the water phase solution product colour changed from light to dark brown was observed which indicates that a certain amount of by-products principally humin compounds were formed. Therefore, the optimized condition of current micro-wave assisted Keggin-type POM catalysis can be assign to 3 mL solvent at 160 °C and 15 min reaction time. Under this optimized condition, 90.1% cellulose conversion with 77.2% glucose yield is achieved.

The above observations clearly indicate excellent cellulose hydrolysis performance by the Keggin-type POM catalyst with an adequate cellulose conversion and glucose yield. The optimization of the catalytic process is highly important as the desired product formed in the system can be further decomposed or polymerized which might decrease the yield. As the Brønsted acidity of polyoxometalates is stronger than typical mineral acids, they are expected to be more efficient catalysts than those of mineral acids for the hydrolysis of cellulose. In a typical study of comparison among different Brønsted acids including heteropolyacids (H_3_PW_12_O_40_ and H_4_SiW_12_O_40_) and mineral acids (HClO_4_, H_2_SO_4_ and H_3_PO_4_) showed that the conversion of cellobiose and the yield of glucose decreased in the following order: H_3_PW_12_O_40_ ≈ H_4_SiW_12_O_40_ > HClO_4_ > H_2_SO_4_ > H_3_PO_4_.^18^ Moreover, they have also reported a glucose yield of ∼50% could be obtained after 24 h of reaction at 393 K using an H_3_PW_12_O_40_ or H_4_SiW_12_O_40_ catalyst. There have been some other previous reports of utilizing the POM-based catalyst for biomass conversion. Using a hydrothermal conversion route, Tian *et al.* achieved glucose yield of 50.5% and selectivity higher than 90% at 453 K for 2 h using 0.42 mass ratio of cellulose to H_3_PW_12_O_40_.^15^ Among the previous literature, the highest cellulose hydrolysis catalytic performance was achieved as glucose yield of 75.6% at 90 °C under microwave irradiation for 3 h using the concentrated H_3_PW_12_O_40_ (88% w/w, 2.63 mol L^−1^).^34^ In our current study, we have achieved glucose conversion of 90.1% with glucose yield of 77.2% using a low concentration of H_3_PW_12_O_40_ (11% w/w, 0.15 mmol), and 15 minutes microwave irradiation which indicates an obvious advancement even better than that of concentrated H_3_PW_12_O_40_ acid in the previous study.^34^

POM contains both Brønsted acidic and metallic part in its structure. Brønsted acidic part makes these compound to behave as strong acid even stronger than the conventional acid such as, SiO_2_–Al_2_O_3_, H_3_PO_4_/SiO_2_, HCl, H_2_SO_4_, HNO_3_*etc.* and thereby exhibit proficient catalytic activity compared to these acids.^18^ These molecules make use of this sites to catalyze the hydrolysis of cellulose.

**Scheme 1:**
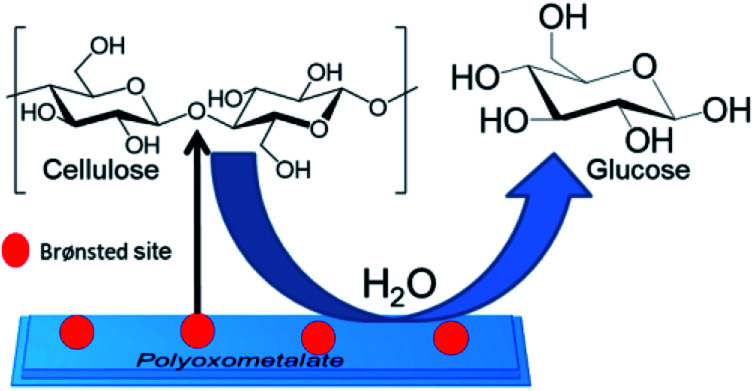
Mechanism of cellulose to glucose conversion using POM catalyst.

Therefore, the general mechanism for the hydrolysis of cellulose using POM catalysis can be described as a simple heterogeneous acid catalysis hydrolysis reaction. The reaction starts with the adsorption of the cellulose on the Brønsted sites of the catalyst and protonates the β-1,4-glycosidic bonds to form a positively charged acyclic or cyclic intermediate. In the final step rapid addition of water molecule results in the formation of glucose molecule with the regeneration of the catalyst for further catalysis (Scheme 1).

The microwave irradiation might play a significant role to accelerate the hydrolysis process using POM-based catalyst. Microwave can be absorbed deeply into both the folding layers of the cellulose to destroy the crystal structures and the POM catalyst that effectively improve the contraction between the catalyst and the solid substrate resulting an efficient hydrolysis is achieved.^22^ The excellent cycle useability of POM catalyst was reported in some previous reports.^15,34^ In particular, after the first catalytic reaction under microwave irradiation, the POM catalyst can be easily recovered from the hydrolytic solution by extraction with diethyl ether. The water-soluble saccharides were not extracted into the solvent layer. Therefore, the separation of the soluble saccharides and the catalyst could be achieved. The recovered POM catalyst could be obtained after the complete evaporation of the diethyl ether, then used for a second run under the same conditions. The efficiency for recyclability was obtained over 90%.^15,34^ Unfortunately, the cycle useability experiment of POM catalysts is beyond our current experimental scope and will include in our upcoming work.

## Conclusions

In summary, the POM clusters were used as microwave-absorbing acid catalysts for the rapid hydrolysis of ball-milling celluloses. Compared to the Wells–Dawson type POM catalyst, the Keggin type POM catalysts show higher performance. The reaction parameters, including temperature, reaction time, and amount of solvent, have a strong influence on the cellulose conversion and glucose yield and are optimized to achieve maximum efficiency under the experimental conditions employed. The optimized conditions for cellulose conversion and glucose yield were estimated at 160 °C for 15 minutes using 3 mL of solvent. In the above-optimized conditions, a remarkably high cellulose conversion of 90.1% and glucose yield of 77.2% were achieved. We believe that the findings from the current study will provide an important future guide to the associated research area.

## Conflicts of interest

There are no conflicts to declare.

## Supplementary Material
